# Nationwide comprehensive human papillomavirus (HPV) genotyping of invasive cervical cancer

**DOI:** 10.1038/s41416-018-0053-6

**Published:** 2018-03-21

**Authors:** Camilla Lagheden, Carina Eklund, Helena Lamin, Sara Nordqvist Kleppe, Jiayao Lei, K. Miriam Elfström, Karin Sundström, Bengt Andrae, Pär Sparén, Joakim Dillner

**Affiliations:** 1 Department of Laboratory Medicine, Karolinska Institutet, 171 77 Stockholm, Sweden; 2 Karolinska University Laboratory, Karolinska University Hospital, 171 76 Stockholm, Sweden; 3 Department of Medical Epidemiology & Biostatistics, Karolinska Institutet, 171 77 Stockholm, Sweden

**Keywords:** Cervical cancer, Tumour virus infections

## Abstract

**Background:**

The Swedish National Cervical Screening Registry collects and evaluates comprehensive, nationwide health data to optimise organised cervical cancer prevention. Since all cervical cancer specimens are saved in biobanks, population-based data from the specimens should be available for analysis and linkage with other health information.

**Methods:**

We identified all cervical cancers diagnosed in Sweden during 2002–2011 (4254 confirmed cases) and requested the tissue blocks to retrieve human papillomavirus (HPV) genotype data using general primer PCR with Luminex genotyping and real-time PCR targeting the *E6/E7* regions of HPV16/18.

**Results:**

We obtained blocks from 2932/4254 (69%) of cases. Valid HPV genotyping data was retrieved for 2850 cases (97%). The most common type was HPV16 (60%), followed by HPV18 (19%), HPV45 (7%), HPV31 (3%), HPV33 (2%), HPV52 (2%), HPV39 (1%), HPV70 (1%), HPV56 (1%), HPV35 (1%), HPV58 (1%) and HPV59 (1%). Ninety-six percent of all HPV-positive cases had a single infection. Eighty-nine cases were HPV-positive only when testing for the HPV16/18-*E6/E7* region.

**Conclusions:**

We present one of the largest series of HPV-genotyped cervical cancers to date. The systematic collection of cervical cancer HPV genotyping data by the screening registry will facilitate prevention and monitoring of HPV type-specific disease burden.

## Introduction

Human papillomavirus (HPV) infection is the major risk factor for cervical cancer.^1^ Although effective screening methods (cytology and HPV testing) exist and several effective prophylactic vaccines are licensed, cervical cancer is still common among women globally.^1^ Research on how effective health services are in real life and methods to obtain an evidence basis for how they could be improved is therefore a priority. Most countries that organise cervical screening programmes also have comprehensive screening registries that can provide the evidence base for evaluation and improvement,^2^ but modes of operation of these registries vary and the rate of innovation to evaluate new methods for data capture and analysis can also be variable.

Sweden has had organised cervical screening since the 1960s, with very high population coverage by international standards (participation rate 82%) (www.nkcx.se); despite this, more than 450 women are diagnosed with cervical cancer every year.^3^ The Swedish National Cervical Screening Registry (NKCx) collects and evaluates comprehensive data on cervical screening, as a basis for evidence-based optimisation of cervical cancer prevention in Sweden.^4^ As part of an initiative to promote excellence in the operation of quality registers in healthcare, the screening registry piloted systematic, population-based HPV genotyping of cervical cancers and importing of resulting data. For all cervical cancers diagnosed in Sweden during a 10-year period, 2002–2011, we requested the corresponding archival diagnostic blocks and subjected the delivered blocks to HPV genotyping. Consecutive and population-based HPV genotyping data of cervical cancers will establish a baseline of the HPV type-specific disease burden as basis for optimising cervical cancer prevention strategies and enable a later comparison after the switch to primary HPV screening and/or implementation of new vaccination strategies.

## Materials and methods

### Data sources and registries used to collect data

**Fig. 1:**
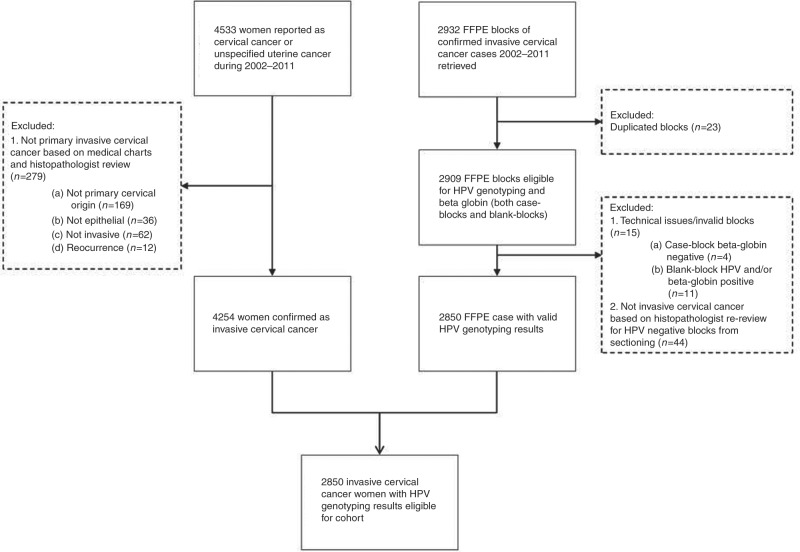
Flowchart

Data on all cases of cervical cancer and unspecified uterine cancer diagnosed between 2002 and 2011 were retrieved from the Swedish National Cancer Registry (NCR). The medical charts of all 4533 registered cancers were retrieved from the respective hospitals and subjected to a detailed review by an experienced gynaecologist. Primary, invasive cancers of epithelial, cervical origin were confirmed in 4254 cases and clinical data including histopathology, FIGO cancer stage and treatment were collected (Fig. 1).

We obtained ethical approval to collect archival material from all cervical cancer cases, to perform histopathology review of the diagnostic slides, and to collect the formalin-fixed paraffin-embedded (FFPE)-blocks for HPV-genotyping. The Regional Ethical Review Board determined that, due to the population-based nature of the study, informed consent from study participants was not required (EPN-Dnr: 2011/1026-31/4) and collection of the samples for histology review and HPV-typing was also allowed (EPN-Dnr: 2012/1028/32).

Access to the samples was requested from 25 different biobanks that serve as regional administrative units. Samples are stored in 30 local pathology laboratories (for Stockholm county the oldest cases are stored in a long-term storage facility). All biobanks approved access to the diagnostic slides. Twenty-two biobanks also approved the application for FFPE-blocks but three biobanks declined. In total, five biobanks never sent any blocks. All diagnostic slides were reviewed by an expert pathologist. In total, 2954 blocks from 2932 patients were received for sectioning. Information on histopathology type was collected and the FFPE block with the highest tumour to healthy tissue proportion and the most severe morphology was chosen for HPV genotyping (one block per patient).

### Sectioning, DNA extraction and genotyping

All cases were sectioned at an accredited laboratory, HistoCenter, Inc. in Gothenburg, Sweden, according to a contamination-proof procedure, as previously described.^5^ The first and last sections for each case were stained with haematoxylin and eosin (H&E-staining) for later re-review. In-between each case-block, a blank-block was sectioned as contamination control. All sections, blank-blocks and case-blocks, were extracted with a xylene-free method^5, 6^ and HPV genotyped using PCR with modified general primers (MGP)-PCR (primer targeting* L1*) and hybridisation with type-specific probes in Luminex, as previously described.^7, 8^ Forty-two beads, encompassing 37 different HPV-types, three HPV variants, and two ‘universal’ HPV probes, were included in the Luminex. If the case was HPV-negative, extracted material from both the blank-block and case-block was diluted 1/10 and re-tested.

Blank-blocks and the matched case-blocks were treated in exactly the same way during the whole process. All samples were analysed for beta-globin by quantitative real-time PCR to confirm sample adequacy, as previously described.^9^ The blank-block had to be negative for both HPV and beta-globin and the case-block positive for beta-globin. Cases that were beta-globin negative were classified as inadequate samples and were not analysed (*n* = 4). Cases with a contaminated blank block were not analysed (*n* = 11). Cases with a valid HPV negative result (both undiluted and diluted 1/10) were re-reviewed by an expert pathologist to confirm presence of invasive cervical cancer tissue, as previously described.^5^ In total, 44 cases were not confirmed to contain invasive cervical cancer tissue in the block and were excluded from the study.

### Quantitative real-time PCR for HPV16 and HPV18

Cases that were HPV-negative in genotyping but whose tumour material was confirmed to contain invasive cervical cancer tissue were analysed for the *E6/E7* regions of the two most common oncogenic HPV types, HPV16 (primer targeting *E7*) and HPV18 (primer targeting *E6*).^5, 10–12^ Briefly, 1 µL DNA was used in both assays with a total volume of 25 µL. Both PCRs used a standard curve with a known concentration of 1–10,000 copies/µL.

### Implications for HPV vaccines

In order to estimate the proportion of the current tumour series that might have been prevented by current HPV vaccines, we calculated the proportion of tumour HPV types covered by the bivalent HPV vaccine, quadrivalent HPV vaccine and nonavalent HPV vaccine, respectively. In this calculation, we only considered single infections and did not consider multiple infections, or potential cross-protection.

## Results

The case ascertainment started with all 4533 cervical cancers registered in the Swedish Cancer Registry over 10 years. Following the detailed medical chart review, 4254 (94%) cases with primary, invasive cancers of epithelial, cervical origin were confirmed. Blocks were obtained for 2932 of 4254 requested blocks (69%). Valid HPV genotyping results were obtained for 2850/2932 cases (97%). The remaining 82/2932 cases (3%) were excluded due to the following reasons: 23 cases because they had more than 1 block, 15 cases because they had no result due to technical issues, and 44 cases because invasive cervical cancer was not confirmed in the re-review of the sectioned tissue block. The HPV genotyping data thus comprised 2850/4254 (67%) of all cervical cancers in the country during the 10-year period investigated.

When considering single infections only, the most common HPV genotype was HPV16 (57% of all HPV-positive cases, 49% of all cases with valid results), followed by HPV18 (19/16%), HPV45 (7.1/6.1%), HPV31 (2.8/2.4%), HPV33 (1.9/1.6%), HPV52 (1.6/1.4%) and HPV39, HPV70, HPV56, HPV35, HPV58 and HPV59 (<1% each). Only one HPV type was present in 2364/2850 (83%) of all cases with valid results. Multiple infections were present in 92/2850 cases (3%) and no HPV infection was detected in 394/2850 cases (14%) (Table 1). Among the 3% of cases with multiple infection, HPV16 was the most commonly occurring type, and all cases with multiple infections had at least one HR HPV type. A low-risk type only (i.e. no HR HPV present) was detected in ~3% of all cases whether considering all cases with valid results or only HPV-positive such (Table 2). When considering HPV type distribution regardless of single and/or multiple infection status, the results remained unchanged compared to when describing type distribution based on single infections only (Table 3).

**Table 1:** HPV status in 2850 cervical cancer cases from a nationwide audit procedure, listed by type (single infections only), multiple, negative respectively, as defined by Luminex and/or RT-PCR results

		% of valid	% of single positive
HPV type	No. of cases (*n*)	% (95% CI)	% (95% CI)
16	1401	49.2 (47.3–51.0)	57.04 (55.1–59.0)
18	459	16.1 (14.8–17.5)	18.69 (17.2–20.9)
45	175	6.1 (5.3–7.1)	7.1 (6.1–8.2)
31	68	2.4 (1.9–3.0)	2.77 (2.2–0.3)
33	46	1.6 (1.2–2.1)	1.87 (1.4–2.5)
52	39	1.4 (1.0–1.9)	1.59 (1.1–2.2)
39	22	0.8 (0.5–1.2)	0.90 (0.6–1.4)
70	22	0.8 (0.5–1.2)	0.90 (0.6–1.4)
56	21	0.7 (0.5–1.1)	0.86 (0.5–1.3)
35	20	0.7 (0.4–1.1)	0.81 (0.5–1.3)
58	18	0.6 (0.4–1.0)	0.73 (0.4–1.2)
59	14	0.5 (0.3–0.8)	0.57 (0.3–1.0)
6	11	0.4 (0.2–0.7)	0.45 (0.2–0.8)
66	8	0.3 (0.1–0.6)	0.33 (0.1–0.6)
53	7	0.2 (0.1–0.5)	0.29 (0.1–0.6)
42	6	0.2 (0.08–0.5)	0.24 (0.09–0.5)
11	5	0.2 (0.06–0.4)	0.20 (0.07–0.5)
67	5	0.2 (0.06–0.4)	0.20 (0.07–0.5)
68	5	0.2 (0.06–0.4)	0.20 (0.07–0.5)
51	4	0.1 (0.04–0.4)	0.16 (0.04–0.4)
73	4	0.1 (0.04–0.4)	0.16 (0.04–0.4)
90	2	0.07 (0.008–0.3)	0.08 (0.01–0.3)
69	1	0.04 (0.001–0.2)	0.04 (0.001–0.2)
87	1	0.04 (0.001–0.2)	0.04 (0.001–0.2)
Multiple	92	3.23 (2.6–3.9)	3.75 (3.0–4.6)
Negative	394	NA	NA
Total	2850	86.2 (84.9–87.4)	100%

**Table 2:** Low-risk HPV type status in cervical cancer cases from a nationwide audit procedure, listed by type (single infections only), as defined by Luminex results

		% of LR
HPV type	No. of cases (*n*)	% (95% CI)
70	22	30.6 (20.2–42.5)
6	11	15.3 (7.9–25.7)
66	8	11.1 (4.9–20.7)
53	7	9.7 (4.0–19.0)
42	6	8.3 (3.1–17.3)
11	5	6.9 (2.3–15.5)
67	5	6.9 (2.3–15.5)
73	4	5.6 (1.5–13.6)
90	2	2.8 0.3–9.7)
69	1	1.4 (0.04–7.5)
87	1	1.4 (0.04–7.5)
Total LR positive	72	100.0%
Total HPV-positive cases	2456	2.9 (2.3–3.7)
Total cases with valid results	2850	2.5 (2.0–3.2)

**Table 3:** HPV type distribution in 2456 HPV-positive cervical cancer cases from a nationwide audit procedure, by prevalence observed. The most common HPV types are listed whether occurring in single and/or multiple infection, respectively

		% of valid	% of positive
HPV type	No. of cases (*n*)	% (95% CI)	% (95% CI)
16 positive	1471	51.6 (49.8–53.5)	59.9 (57.9–61.8)
18 positive (16 negative)	469	16.5 (15.1–17.9)	19.1 (17.6–20.7)
45 positive (16 and 18 negative)	180	6.3 (5.5–7.3)	7.3 (6.3–8.4)
31 positive (16, 18 and 45 negative)	69	2.4 (1.9–3.1)	2.8 (2.2–3.5)
33 positive (16, 18, 31 and 45 negative)	47	1.6 (1.2–2.2)	1.9 (1.4–2.5)
52 positive (16, 18, 31, 33 and 45 negative)	42	1.5 (1.1–2.0)	1.7 (1.2–2.3)
Other HR (16, 18 and 45 negative)	106	3.7 (3.1–4.5)	4.3 (3.5–5.2)
LR (single infections only)	72	2.5 (2.0–3.2)	2.9 (2.3–3.7)
Total	2456	86.2 (84.9–87.4)	100%

The slides from the sectioning were re-reviewed by the study pathologist for the 527/2850 (18%) of cases that were negative after HPV genotyping. No invasive cervical cancer tissue was found in 44/527 (8%) of these cases and therefore, they were excluded after this re-review. The remaining 483/527 (92%) negative cases were analysed for HPV16 (*E7* gene) and HPV18 (*E6* gene) using quantitative real-time PCR. Eighteen percent (89/483) of these cases were positive for *E6/E7* of HPV16 and/or HPV18.

When combining the data on single infections from the HPV genotyping and real-time PCR, the two HPV types (HPV16 and 18) in the bivalent vaccine were found in 79% of all HPV-positive (65% of all) cervical cancers. The proportion of tumours positive for the four types in the quadrivalent vaccine (HPV6, 11, 16 and 18) was also 79%. The types in the nonavalent HPV vaccine (HPV6, 11, 16, 18, 31, 33, 45, 52 and 58) were found in 94% of all HPV-positive cases and 78% of all cases (Table 4).

**Table 4:** Potential vaccine protection obtained from currently approved HPV vaccines, estimated using information from HPV single type infections

		% of valid	% of positive
Vaccine types	No. of cases (*n*)	% (95% CI)	% (95% CI)
Bivalent (HPV16 and 18)	1860	65.3 (63.5–67.0)	78.7 (77.0–80.3)
Quadrivalent (HPV6,11, 16 and 18)	1876	65.8 (64.1–67.6)	79.4 (77.7–81.0)
9-valent (6, 11, 16, 18, 31, 33, 45, 52 and 58)	2222	78.0 (76.4–79.5)	94.0 (93.0–94.9)

## Discussion

We report that systematic and nationwide HPV genotyping of a consecutive series of invasive cervical cancers in a nationwide case-control audit is feasible to perform as part of the routine programme improvement and quality assurance work of a cervical screening registry. In so doing, we provide one of the largest series of HPV genotyping data in cervical cancer to date.

We used identifiable data from the comprehensive National Cancer Registry and had access to medical records for case ascertainment and to a majority of the archival blocks from the pathology biobanks in the country.

Contamination-proof, standardised and quality-assured sectioning of FFPE blocks is an important bottleneck in many studies of viral detection in FFPE specimens and we were fortunate to locate a certified sectioning company that could provide this. The review of all slides by an experienced pathologist to both confirm invasive epithelial cervical cancer and to select which block should be sectioned and tested (based on highest proportion of invasive tissue in the block) is another important strength. The alternative strategy to select the block that tests positive in the case of multiple blocks creates a systematic bias and should be avoided.

Although all pathology biobanks were (in principle) open access, not all blocks from case patients were actually sent. A number of blocks were not sent due to quality issues such as lack of tissue left in the block, or the diagnostic slides we required for review having been discarded. Furthermore, 636/4275 blocks (~15%) were not sent because the biobank did not approve our application for access. Three of the 22 counties approved the application but did not send any blocks. Some offered to instead provide sections from the block if they performed the sectioning themselves. As mandated by European Union purchasing laws for services worth as much as the amount of sectioning required by this study, we requested sectioning services through a formalised purchasing procedure and were therefore legally unable to use other sectioning services than from the company that had won the tender. Also, a central, accredited laboratory for sectioning is crucial in order to avoid variability in sectioning procedures. Ordinary H&E staining of the first and last section before the sections that were HPV genotyped enabled verification of whether the sections tested did indeed contain cancer tissue.

Limitations of our study include that some diagnostic FFPE-blocks might have been sectioned so many times that no cancer tissue is left in the block that was delivered to us. Pathologist services to re-review the entire study of almost 6000 slides, first and last for all cases, was too expensive and we therefore decided, already at the outset, to re-review only HPV-negative specimens. This could conceivably have resulted in that some positive HPV-tests derive from specimens where cancer tissue was not present. However, only a small proportion (8%) of the HPV-negative specimens that we did re-review were excluded because of lack of cervical cancer tissue and since specimens positive for a virus with tropism for epithelium should have contained at least some epithelial tissue, the proportion of false positives is likely to be even lower in the full material. The fact that very few tumour specimens contained more than one HPV type also suggests that presence of HPV types originating not from the cancer but from adjacent benign epithelium is likely to be very low.

Some cervical cancers in this study were HPV DNA negative, even though HPV is considered a necessary risk factor and virtually all cervical cancers in situ are HPV-positive.^13^ Cervical cancer cases that appear to have lost HPV DNA seem to constitute a specific subgroup of cervical cancers with a different biological behaviour (worse prognosis).^14, 15^ Possibly, some or all of the HPV genome may have been lost with increasing progression of the tumour resulting in that the HPV *L1* region was no longer detectable. Indeed, the proportion of HPV-positive cancers increased when also testing for the *E6/E7* region. Future efforts should be directed at testing whether parts of the HPV genome may still be present in these apparently HPV-negative cancers.

When estimating the effect of eliminating HPV vaccine types from the population, the proportion of all cervical cancers that were found to contain vaccine types (e.g 78% containing the nine vaccine types included in the nonavalent vaccine) constitutes a lower bound of the estimated effect and the proportion of HPV-positive cancers that contain vaccine types (e.g. 94% for the nonavalent vaccine) constitutes an upper bound of the estimated effect. A large meta analysis^16^ found that more than 2/3 of ICC and half of HSIL could be prevented by vaccines protecting against HPV16 and 18, a finding which we confirm.

Cross protection seems to vary between the vaccines and elimination of HPV types merely by cross-protection is conceivable,^17^ but estimation of the possible effects of cross-protection was considered to be beyond the scope of this paper. Similarly, formal predictions of number of cases prevented for each year after vaccination would require a more comprehensive age and period effect analysis. The problem with assigning causality on cases with multiple infections has been extensively discussed and several strategies to approach the problem have been proposed.^18^ However, none of these approaches are entirely satisfactory. Therefore, as 96% of our cases were only positive for a single HPV type, and consideration of multiple infections did not change which conclusions on type distribution, we decided to focus our analysis on single infections only.

### Conclusion

We present one of the largest series of HPV-genotyped cervical cancers to date. The systematic collection of cervical cancer HPV genotyping data by the screening registry will facilitate prevention and monitoring of the HPV type-specific disease burden.

## Availability of data and material

Individual-level data will be shared on request, to be sent to the corresponding author, given that all ethical and legal requirements of such a transfer are met.
